# Noninvasive Evaluation of Liver Fibrosis in Patients with Chronic Hepatitis C

**DOI:** 10.5812/kowsar.1735143X.738

**Published:** 2011-09-01

**Authors:** Jose M. Ladero

**Affiliations:** 1 Liver Unit, Gastroenterology Service, Hospital Clinico San Carlos, Department of Medicine, Complutense UniversityMadrid, Spain

**Keywords:** Liver fibrosis, Chronic hepatitis C

|Only 20% of patients with chronic hepatitis C (CHC) progress to cirrhosis [1]. Progressive fibrosis is the hallmark of an unfavorable course and is related to several factors such as male gender, contracting the disease at an older age, features of the metabolic syndrome, iron overload, ethanol abuse, and co-infection with hepatitis B or human acquired immunodeficiency viruses. However, the course of the disease is highly variable from individual to individual. Liver biopsy remains the gold standard for evaluating necroinflammation (grade) and fibrosis (stage) in CHC [2]; however, this pre-eminence is currently being examined due to its intrinsic risks and limitations. Liver biopsy is an invasive procedure that carries a risk of complications, although rarely severe. The biopsy provides a very small specimen of liver tissue and has both a significant rate of sampling error, especially when the cylinder obtained is less than 25 mm in length, and interobserver variability. The risk-benefit ratio of liver biopsy is insufficient to maintain it as a firstline procedure, and new and non-invasive criteria for the evaluation of liver fibrosis are urgently needed [3].

A discussion on the molecular pathobiology of liver fibrogenesis is beyond the scope of this article (for a review see references [4] and [5]. The ideal marker for liver fibrosis must be specific to fibrosis of the liver, not influenced by co-morbidities, sensitive, reproducible, and informative for both the current stage of fibrosis as well as the rate of fibrogenesis activity [6]. For practical purposes, non-invasive fibrosis markers can be classified as direct (Class I biomarkers), indirect (Class II biomarkers), and imaging methods [7].

## 1. Classifications of Noninvasive Fibrosis Markers

### 1.1. Class I biomarkers

These reflect the greater deposition of extracellular matrix in the liver due to either increased synthesis by activated stellate cells (ASC) or slow removal by Kupffer and endothelial sinusoidal cells. They translate to an imbalance between the synthesis and degradation of collagen, but they do not reflect the current stage of fibrosis. Many of these substances are collagen components or by-products, extracellular matrix-related enzymes, glycoproteins and matrix metalloproteinases, or glycosaminoglycans. However, this category also includes some growth factors such as transforming growth factor β (TGFβ) and connective tissue growth factor [4]. The cost and complexity of these determinations and their relatively low specificity and sensitivity greatly reduce their clinical usefulness. This has been the case for type I, III, and IV procollagens and for most enzymes involved in the synthesis and degradation of extracellular matrix. However, 3 possible candidates for clinical use can be cited:

a) Hyaluronic acid (HA): Plasma levels of this glycosaminoglycan, which is synthesized by HSC and degraded by liver sinusoidal cells, are increased in CHC due to delayed degradation, and correlate with the stage of fibrosis [8]. Its main value is to exclude advanced fibrosis or cirrhosis; however, it losses much of its usefulness in distinguishing among earlier stages of fibrosis. It has been proposed as a component of some scoring indexes, as discussed under class II biomarkers.

b) Glycoprotein YKL-40 (chondrex) is a fibroblast and endothelial growth factor that belongs to the chitinase family. YKL-40 has shown good specificity (81%) and sensitivity (78%) for distinguishing between low-null (F0-F1) and significant (F2-F4) fibrosis in CHC. However, YKL-40 is not liver-specific and may be elevated in extrahepatic diseases, such as arthritis [9].

c) Connective tissue growth factor (CTGF, CCN2) is synthesized by activated HSC and hepatocytes under differential stimulus by TGFβ and is up-regulated in the earlier and intermediate stages of collagen synthesis, but decreases (as does its main stimulus, TGFβ) when cirrhosis is established. Therefore, CTGF could be a good marker of ongoing fibrogenesis, but not of cirrhosis [4].

### 1.2. Class II biomarkers

They are empirically defined combinations of biochemical tests (and sometimes platelet counts) reflecting liver changes that are induced, at least is part, by fibrosis, but are not directly related to the mechanisms of fibrogenesis. The obligate gold standard in the histological staging of fibrosis and the passport to their existence beyond the original report is a confirmed satisfactory AUROC (area under receiver operator curve) in independent studies. Single data, such as low a platelet count, high GGT plasma level, or a GOT/GPT quotient > 1, are well known as surrogate markers of advanced liver disease and thus of significant or severe fibrosis. In a recent review of 9 scoring methods [10], we have not found substantial advantages among the most efficient ones (APRI, GUCI, King's, and FIB-4). In fact, the most well known and easier to calculate method, APRI, is not worse than the newer and more complex King's score. At least in our experience, Forns' score is the most sensitive for distinguishing between intermediate stages of fibrosis. Our personal contribution to this field was the finding that when added to APRI and King's scores, serum ferritin concentration improved the accuracy for detecting null-low fibrosis, and thus reduced the need to perform a liver biopsy. However, this feature needs independent confirmation.

Other more sophisticated and expensive methods are those that include in their formulas data that are not available as standard laboratory tests. The most well known in this category is FibrotestTM (FibrosureTM in the USA), a commercially available test that is calculated by centrally introducing into its equation values for haptoglobin, α2-macroglobulin, apolipoprotein A1, GGT, and bilirubin. Fibrotest promoters [11], as well as several independent studies, claim that Fibrotest is better than APRI for determining the intermediate stages of fibrosis; however, it loses part of its efficacy in patients with normal aminotransferase levels [7]. Other scoring methods that include non conventional parameters are the ELF score (PIIINP, hyaluronic acid, and TIMP-1) [12] and FIBROSpect II, Fibrometer, and Hepascore, which include several class I biomarkers, but still have unproven superiority over the simpler scores, such as APRI [5].

It has been proposed that the combination or the sequential use of several scoring systems, in association with Fibroscan, if necessary (see later), could improve their diagnostic accuracy. Several sequential algorithms that combine APRI, Forns' index, and Fibrotest provide high specificity in detecting F0-F1 fibrosis or cirrhosis, depending on the cut-off points selected, thus reducing the need to perform liver biopsies in a substantial proportion of patients [6].

### 1.3. Imaging tests

Ultrasonography (US): Several features of ultrasonography provided by standard US studies, such as portal venous flow and portal flow velocity, may be useful for the diagnosis of established cirrhosis. However, they are unable to discriminate between less advanced stages of fibrosis with a minimum precision, and it is usual to obtain a normal US study in a patient with advanced fibrosis.

Transient elastography (FibroScan®): Fibrosis increases hepatic rigidity and this method provides a measure of hepatic stiffness that is roughly proportional to the stage of cirrhosis. FibroScan is innocuous and easy to perform. It evaluates a virtual section of the liver that is much greater than the minimal cylinder provided by the needle biopsy (thus reducing the risk of sampling error), and better accuracy than several scoring systems has been shown in most, if not all, published comparative studies. However, this method is operator-dependent and has intrinsic technical limitations in both its performance and adequate interpretation (i.e. narrow intercostal spaces, obesity, or steatosis). FibroScan is an excellent tool when the clinical problem is to exclude cirrhosis (as are most of the scoring systems based on type II biomarkers); but, it losses sensitivity and specificity when the aim is to differentiate between less advanced stages of fibrosis [13]. In spite of its wide acceptance among both physicians and patients, the clinical usefulness of transient elastography is far from firmly established, and it cannot be currently proposed as an alternative for liver biopsy in the detection of significant fibrosis (METAVIR ≥ F2), which is the main criterion used by most clinicians to make the decision for antiviral treatment [14].

Magnetic resonance imaging (MRI): Novel MRI applications have allowed for the detection of pathological abnormalities in liver diseases. The sequential administration of gadolinium and super magnetic iron oxide (SPIO) contrasts with adequate software analysis has provided highly valuable patterns of fibrosis compared with histopathological features [15]. Preliminary results with diffusion-weighted MRI and MRI spectroscopy are promising, as are studies with MR Elastography. However, MRI equipment and performance are costly, uncomfortable for patients, and time consuming, thus reducing the probability that MRI will have a significant role in liver fibrosis evaluation [16].

## 2. New developments

The search for new and more reliable non-invasive serological biomarkers of liver fibrosis has found an excellent allied in proteomics. Ho et al. [17] detected 3 putative biomarkers of fibrosis in chronic hepatitis C: α-2 macroglobulin (up-regulated), vitamin D binding protein (VDBP) (down-regulated), and apolipoprotein A1 (downregulated). In a recent study in our laboratory, we have confirmed the presence of low levels of VDBP (fraction 3) in patients with hepatitis C and low stages of fibrosis when compared with normal controls (manuscript in preparation). Proteomic methods are continuously being improved [18], and it is to be expected that they will provide us with new and powerful markers of fibrosis not only in chronic hepatitis C, but in a wide range of chronic liver diseases.
